# Diagnostic delay, *SERPING1* allelic heterogeneity, and real-world lanadelumab prophylaxis in Chinese patients with hereditary angioedema due to C1 inhibitor deficiency

**DOI:** 10.3389/falgy.2026.1899305

**Published:** 2026-08-05

**Authors:** Wenjin Du, Siqin Wang, Ke Yang, Qiuxing Zhang, Xianghua Lin, Wenchao Zhang, Weili Guo

**Affiliations:** 1 Department of Allergy, Henan Provincial People’s Hospital, People’s Hospital of Zhengzhou University, People’s Hospital of Henan University, Zhengzhou, Henan, China; 2 Henan Key Laboratory of Genetic Diseases and Functional Genomics, Medical Genetics Institute, Zhengzhou University People’s Hospital, People’s Hospital of Henan University, Zhengzhou, Henan, China

**Keywords:** C1 inhibitor deficiency, China, diagnostic delay, hereditary angioedema, lanadelumab, long-term prophylaxis, real-world evidence, *SERPING1*

## Abstract

**Background:**

Hereditary angioedema due to C1 inhibitor deficiency (HAE-C1INH) is a rare and potentially life-threatening bradykinin-mediated disorder most commonly associated with pathogenic variants in *SERPING1*. Data from Chinese patients remain limited, particularly regarding diagnostic delay, *SERPING1* allelic heterogeneity, and real-world use of lanadelumab prophylaxis.

**Methods:**

In this single-center retrospective observational study, we reviewed 10 consecutive unrelated index patients with confirmed HAE-C1INH managed at Henan Provincial People’s Hospital from January 2022 to May 2026. Clinical data, complement results, and *SERPING1* Sanger sequencing findings were analyzed. Variants were classified according to ACMG/AMP criteria. Seven patients received lanadelumab long-term prophylaxis. Annualized attack rates and Angioedema Control Test scores before and during prophylaxis were compared using the Wilcoxon signed-rank test.

**Results:**

The cohort included 6 females and 4 males, with a mean age of 32.8 ± 9.9 years. Nine patients had HAE-C1INH type 1 and one had type 2. The mean age at symptom onset was 21.7 ± 11.1 years, and the mean diagnostic delay was 9.4 ± 7.0 years. All patients had recurrent peripheral edema, 7 had abdominal attacks, and 6 had facial and/or laryngeal involvement. One patient required emergency tracheotomy. Serum C4 was reduced during attacks in all patients, and C1q levels were normal. Ten distinct heterozygous *SERPING1* variants were identified, including 3 frameshift variants, 6 missense variants, and 1 in-frame deletion. Among the 7 patients receiving lanadelumab, the median annualized attack rate decreased from 11 (IQR, 7–23) to 0 (IQR, 0–1) attacks/year, and the median AECT score increased from 3 (IQR, 2–4) to 13 (IQR, 13–13). No serious adverse events were recorded.

**Conclusions:**

This single-center retrospective study showed substantial diagnostic delay, marked *SERPING1* allelic heterogeneity, and favorable real-world outcomes with lanadelumab prophylaxis in Chinese patients with HAE-C1INH. Earlier complement testing and access to appropriate genetic testing may shorten diagnostic delay. Larger prospective studies are needed to define individualized long-term prophylaxis strategies in Chinese patients.

## Background

Hereditary angioedema is a rare autosomal dominant disorder characterized by recurrent, unpredictable, non-pruritic, non-pitting swelling of the skin, gastrointestinal tract, and upper airway (1). Population-based studies estimate its prevalence at approximately 1.0–1.6 per 100,000 individuals (2–4). Although awareness of the disease has improved, clinical and genetic data from Chinese patients remain limited (5, 6).

Most cases are caused by heterozygous pathogenic variants in *SERPING1*, which encodes C1 inhibitor, a serine protease inhibitor involved in the regulation of complement, contact activation, coagulation, and fibrinolytic pathways (7, 8). Deficiency or dysfunction of C1 inhibitor leads to increased plasma kallikrein activity, excessive bradykinin generation, and vascular leakage (1, 9).

HAE-C1INH is classified into type 1 and type 2. Type 1 is characterized by reduced C1 inhibitor antigen and function, whereas type 2 shows normal or increased antigen level with reduced function (7–9). Type 1 is reported to be more common than type 2 in HAE-C1INH cohorts globally (5). In our cohort, 9 of 10 patients (90%) had type 1 and 1 patient (10%) had type 2, consistent with established epidemiological patterns (Table 1). Some patients exhibit typical clinical features with normal C1INH levels, a genetically distinct condition known as HAE with normal C1INH (HAE-nC1INH). A subset of HAE-nC1INH patients has been found to carry pathogenic variants in *F12*, *PLG*, *ANGPT1*, *KNG1*, *MYOF*, *HS3ST6*, *CPN*, *DAB2IP*, and other genes (9–12); however, such variants are identified in only a limited proportion of clinically suspected HAE-nC1INH cases.

**Table 1:** Clinical and laboratory characteristics of 10 patients with HAE-C1INH.

Characteristic	Patient									
	1	2	3	4	5	6	7	8	9	10
Sex	Female	Male	Male	Female	Female	Male	Female	Female	Female	Male
C4 (g/L)	0.04	0.02	0.05	0.02	0.07	0.05	0.07	0.05	0.07	0.05
C1INH antigen (g/L)	0.03	0.07	0.11	0.70	0.05	0.04	0.08	0.08	0.05	0.11
C1INH function (%)	4.2	4.3	2.5	6.3	13.5	6.6	13.1	21.4	11.0	14.7
C1q (mg/L)	188.0	222.36	179.35	204.46	163.44	174.28	193.86	217.98	166.55	191.22
HAE-C1INH type 1/type 2	type 1	type 1	type 1	type 2	type 1	type 1	type 1	type 1	type 1	type 1
Family history	Yes	Yes	No	Yes	No	Yes	Yes	Yes	Yes	Yes
Current age (years)	18	36	27	38	19	46	44	36	22	42
Age at onset (years)	11	26	22	16	7	35	39	32	10	19
Diagnostic delay (years)	5	8	4	20	10	7	4	2	11	23
Sites of edema	Limbs, abdomen, face, larynx	Limbs, abdomen	Limbs	Face, larynx, limbs, abdomen	Face, larynx, limbs	Limbs, abdomen	Face, larynx, limbs	Limbs, abdomen, face, larynx	Limbs, abdomen, perineum, buttocks	Face, larynx, limbs, abdomen
Triggers	Emotional stress	Fatigue	Fatigue, menstruation, trauma	Cold exposure, emotional stress	Cold exposure, emotional stress	No obvious trigger	No obvious trigger	Fatigue, menstruation	Menstruation, emotional stress	No obvious trigger
Attack frequency	4–5/year	1–3/month	Irregular	7–8/year	2–3/month	Every months	Every 2 months	1–2 every 2 months	Irregular	1–2/month
Duration of attacks (days)	5–7	2–3	1–2	2–5	3–4	2–3	4–5	2–4	3–4	3–5
Severity of attacks	Moderate; abdominal pain, occasionally bedridden	Moderate; abdominal pain requiring bed rest	Mild	Severe; facial and laryngeal edema	Severe; facial and laryngeal edema	Mild	Severe; facial and laryngeal edema	Severe; required bed rest	Severe; abdominal pain requiring bed rest	Severe; previously treated with fresh frozen plasma and tracheotomy
Laryngeal edema	Yes	No	No	Yes	Yes	No	Yes	Yes	N0	Yes
Comorbidities	OCD	HBV	None	None	None	Allergic asthma	None	Anemia	Drug allergy (amoxicillin)	Pulmonary tuberculosis
Current LTP	Untreated	Lanadelumab	Untreated	Lanadelumab	Lanadelumab	Lanadelumab	Lanadelumab	Lanadelumab	Icatibant (Acute rescue therapy)	Lanadelumab

Reference ranges: C4, 0.10–0.40 g/L; C1INH antigen, 0.21–0.39 g/L; C1INH function, ≥ 68.0%; C1q, 159–233 mg/L.

HAE, hereditary angioedema; C1INH, C1 inhibitor; HBV, hepatitis B virus; OCD, obsessive-compulsive disorder; VAS, visual analogue scale.

More than 850 *SERPING1* variants have been described, including missense, frameshift, splice-site, nonsense, and large rearrangement variants (8, 13). Most are private variants, reflecting marked allelic heterogeneity. Approximately 30%–34% arise *de novo*, challenging diagnosis in patients without family history (14). In patients with a strong clinical suspicion but negative conventional exon-based *SERPING1* sequencing, deep intronic variants and copy-number variants may require additional methods for detection (15–18).

Due to rarity and non-specific manifestations, HAE is frequently misdiagnosed as allergic angioedema, urticaria, or acute abdomen, causing substantial diagnostic delay and morbidity (19). International surveys report mean diagnostic delays of ∼12.6 years, with 65% experiencing misdiagnosis and 24% undergoing unnecessary abdominal surgery (20). Prolonged diagnostic delays have also been observed in Asia-Pacific regions (6). Early recognition through complement testing and genetic analysis is critical (9, 21).

Lanadelumab, a fully human monoclonal antibody targeting plasma kallikrein, is recommended as first-line long-term prophylaxis (LTP) (9). The HELP trials demonstrated marked attack reduction and favorable tolerability (22–24). Real-world studies from diverse populations, including emerging Asian data, support cross-ethnic effectiveness (25–29). However, additional real-world data from Chinese patients remain limited.

We conducted this single-center retrospective observational study to characterize diagnostic delay, clinical features, complement profiles, and *SERPING1* variant diversity in 10 unrelated Chinese patients with HAE-C1INH, and to describe preliminary real-world lanadelumab outcomes.

## Methods

### Study design and participants

**Figure 1:**
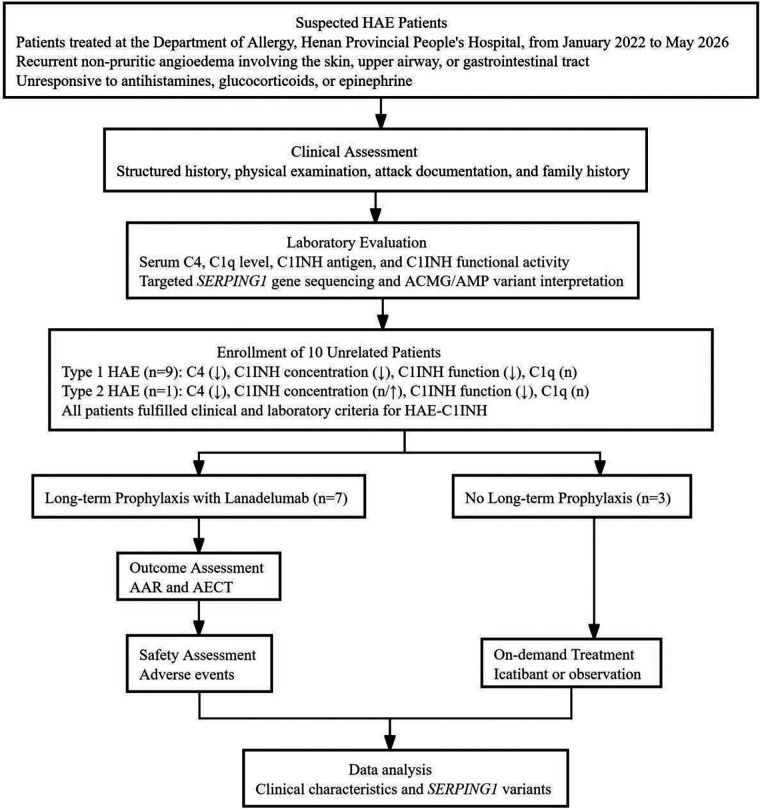
Study flow diagram of patient enrollment, classification, and treatment. This flow diagram illustrates the process of patient screening, diagnostic evaluation, enrollment, treatment allocation, and outcome assessment in this study. “↓” indicates decreased levels of C1INH antigen, C1INH function, or C4; “n” indicates normal levels of C1INH antigen or C1q; “↑” indicates increased levels of C1INH antigen. AAR, annualized attack rate; AECT, angioedema control test; C1INH, C1 esterase inhibitor; HAE, hereditary angioedema.

This single-center retrospective observational study included 10 consecutive unrelated index patients with confirmed HAE-C1INH who received care at the Department of Allergy, Henan Provincial People’s Hospital, from January 2022 to May 2026 (Figure 1). Eligible patients were unrelated index cases with a clinical history compatible with recurrent bradykinin-mediated angioedema and laboratory evidence of C1INH deficiency or dysfunction. HAE-C1INH was diagnosed according to the 2021 WAO/EAACI guideline (9) and the Chinese Expert Consensus (30).

Acquired C1INH deficiency was excluded based on clinical context, normal C1q levels, absence of anti-C1INH antibodies tested only in the two index patients without a family history; see below (detected by indirect enzyme-linked immunosorbent assay [ELISA]), and lack of features suggestive of lymphoproliferative or autoimmune disease. Patients with angioedema associated with chronic spontaneous urticaria, drug-induced angioedema such as ACE inhibitor-associated angioedema, or isolated allergic angioedema responsive to antihistamines were excluded. Clinical data were obtained from electronic medical records and telephone follow-up. Recorded variables included sex, age, age at symptom onset, diagnostic delay, family history, edema sites, triggers, attack frequency, attack duration, comorbidities, prior misdiagnoses, laboratory results, genetic findings, and treatments. The final follow-up was completed in May 2026. The study was approved by the Ethics Committee of Henan Provincial People’s Hospital (No. 2022-2211273107). Written informed consent for genetic testing and publication of anonymized clinical and genetic data was obtained from all participants.

### Complement and C1INH measurements

Peripheral venous blood (2 mL) was collected at the time of clinical presentation. Because all index patients were confirmed clinically during acute angioedema episodes, blood samples were obtained during these acute attacks; therefore, the laboratory results reported in Table 1 represent acute-phase measurements. Most index patients were referred to and first assessed at our tertiary center during an acute attack—typically the abdominal or laryngeal event that prompted specialist referral—so that the initial confirmatory sampling was obtained during the acute phase. This reflects the referral pattern of a tertiary center rather than an absence of interval diagnosis; symptomatic first-degree relatives identified through family history were advised to undergo interval (non-acute) complement screening and cascade genetic testing. Repeat complement measurements during remission were obtained when clinically feasible to assess whether abnormalities persisted beyond the acute phase. Serum C4, C1q, and C1INH antigen levels were determined by nephelometry using the immunoturbidimetric method (scatter nephometry) (reference ranges: C4, 0.10–0.40 g/L; C1INH antigen, 0.21–0.39 g/L; C1q, 159–233 mg/L). C1INH functional activity was assessed using a chromogenic substrate assay (reference range, ≥ 68%). Anti-C1INH antibodies were tested by indirect enzyme-linked immunosorbent assay (ELISA) only in the two index patients without a family history (Patients 3 and 5), in whom acquired C1INH deficiency required exclusion despite normal C1q levels; antibody testing was not performed routinely in the eight patients with a positive family history. Although normal C1q makes acquired deficiency unlikely, C1q may remain normal in a minority of acquired cases, so antibody testing was used as a confirmatory step in these sporadic cases only. When possible, abnormal complement results were confirmed by repeat testing. Assays were performed in the hospital clinical laboratory according to standardized operating procedures. HAE-C1INH type 1 was defined by reduced C1INH antigenic level and reduced functional activity, whereas HAE-C1INH type 2 was defined by normal or elevated C1INH antigenic level with reduced functional activity.

### DNA extraction and *SERPING1* sequencing

HAE-C1INH was diagnosed on clinical and complement (biochemical) grounds according to the 2021 WAO/EAACI guideline; *SERPING1* sequencing was performed for research and cascade-screening purposes and was not required for clinical diagnosis. Genomic DNA was extracted from 2 mL of peripheral whole blood using the DNeasy Blood & Tissue Kit (Qiagen, Hilden, Germany; Cat. No. 69504) according to the manufacturer’s instructions. Primers targeting the *SERPING1* promoter region, all eight exons, and flanking intronic regions were designed using Primer3 (https://bioinfo.ut.ee/primer3-0.4.0/). PCR amplification was performed under standard conditions for 35 cycles. PCR products were purified and sequenced bidirectionally using an ABI 3500 Dx Genetic Analyzer (Applied Biosystems, Foster City, CA, USA).

Sequence data were aligned to the *SERPING1* reference transcript NM_000062.3, and variant nomenclature followed Human Genome Variation Society recommendations. Primer sequences and corresponding amplicon sizes are available upon reasonable request. Because Sanger sequencing was limited to the *SERPING1* promoter, coding exons, and flanking intronic regions, large deletions or duplications, deep intronic variants, and other copy-number variants may not have been detected.

### Variant annotation and classification

Variants were annotated using ClinVar, LOVD, HGMD, and gnomAD v4.1. In silico prediction tools included REVEL for missense variants, MutationTaster/AutoPVS1 for frameshift variants, and SIFT Indel for in-frame deletions. Variant classification was independently performed by two investigators according to ACMG/AMP criteria (31), and discrepancies were resolved by consensus. Variants of uncertain significance were interpreted in the context of clinical phenotype, complement results, family history, and segregation data when available.

### Lanadelumab prophylaxis and outcomes

Seven patients received lanadelumab long-term prophylaxis after shared decision-making. The initial dose was 300 mg subcutaneously every 2 weeks (q2w). Dosing intervals were individualized and extended in selected patients meeting either of the following criteria sustained over ≥6 months: (1) attack-free status, defined as zero angioedema episodes during the preceding 6-month observation period; or (2) well-controlled disease, defined as an AECT score ≥10 with no more than one mild breakthrough attack per year that did not require acute rescue treatment. Extension was not protocol-driven but individualized based on clinical judgment and patient-specific factors. Post-treatment AAR was calculated as the number of attacks during lanadelumab prophylaxis divided by the duration of lanadelumab exposure in years.

Clinical outcomes included: (1) AAR, defined as the number of attacks per year at baseline, based on the 12 months before lanadelumab initiation, and during the prophylaxis period, annualized according to the duration of lanadelumab exposure; (2) disease control, assessed by the AECT (score range, 0–16; ≥10 indicating well-controlled disease) at baseline, based on the 3-month period before treatment, and at the most recent 3-month follow-up; and (3) safety outcomes, including breakthrough attacks, injection-site reactions, serious adverse events, thromboembolic events, and treatment discontinuation. Breakthrough attacks were defined as angioedema episodes occurring during lanadelumab prophylaxis, irrespective of whether rescue medication was used. During the study period (January 2022–May 2026), multiple on-demand treatments were available in China for acute HAE-C1INH attacks, including: (1) icatibant (30 mg subcutaneous injection); (2) fresh-frozen plasma (FFP); and (3) attenuated androgens (danazol) in selected cases. As documented in Table 1, Patients 1 had previously received FFP. Patient 9 received subcutaneous icatibant 30 mg for an acute abdominal attack.

### Statistical analysis

Statistical analyses were conducted using SPSS version 20.0 (IBM Corporation, Armonk, NY, USA). Normality was assessed with the Shapiro–Wilk test. Normally distributed continuous variables are expressed as mean ± standard deviation (SD), and non-normally distributed variables are expressed as median (interquartile range [IQR]). Categorical variables are presented as counts and percentages. Given the small sample size and paired design, pre- and post-treatment AAR and AECT scores were compared using the exact two-sided Wilcoxon signed-rank test. No formal power calculation was performed because of the exploratory nature of the study and the limited sample size. No adjustment for multiple comparisons was applied. *P*-values were interpreted descriptively rather than as confirmatory evidence.

## Results

### Baseline clinical characteristics

Baseline demographic and clinical data are summarized in Table 1. The cohort included 6 females and 4 males. Mean age at last follow-up was 32.8 ± 9.9 years, ranging from 18 to 46 years. Nine patients had HAE-C1INH type 1 and one had type 2. Eight patients had a positive family history, whereas Patients 3 and 5 had no known family history.

Mean age at symptom onset was 21.7 ± 11.1 years. Median age at onset was 20.5 years, with a range of 7–39 years. Mean diagnostic delay was 9.4 ± 7.0 years, and the longest delay was 23 years.

All patients had recurrent peripheral edema. Seven patients had abdominal attacks, and 6 patients had facial/laryngeal involvement. Attacks were non-pitting, non-pruritic, and asymmetric. Most episodes resolved spontaneously within 2–7 days.

Reported triggers included emotional stress, fatigue, cold exposure, menstruation, minor trauma, and prolonged standing. Three patients had no identifiable trigger.

Before appropriate diagnosis or treatment, all patients had received oral antihistamines without benefit. One patient had been treated for chronic urticaria. Five patients with abdominal symptoms had been hospitalized for suspected pancreatitis, appendicitis, or gastroenteritis. Two received fresh-frozen plasma with clinical improvement. Patient 10 had severe laryngeal edema requiring emergency tracheotomy and had previously received fresh-frozen plasma with clinical improvement.

Comorbidities included obsessive-compulsive disorder, chronic hepatitis B, allergic asthma, anemia, drug allergy to amoxicillin, and pulmonary tuberculosis. Based on available clinical records and annualized estimates before treatment, the mean attack frequency was 13.4 ± 8.3 attacks/year. The mean untreated attack duration was 3.4 ± 1.3 days, and the mean peak VAS score was 6.9 ± 2.5.

### Complement and C1INH results

Serum C4 was reduced in all 10 patients during attacks, with a mean value of 0.049 ± 0.019 g/L (reference 0.10–0.40 g/L). Because all patients were confirmed during acute attacks, the C4 values reported in Table 1 represent acute-phase measurements. To address whether complement reduction was transient (acute consumption artifact) or persistent, repeat complement measurements during remission were obtained when clinically feasible. Remission-phase sampling was available for 6 patients (Patients 1, 2, 4, 6, 8, and 10). C4 remained reduced during remission (mean 0.061 ± 0.015 g/L), and the acute-phase versus remission-phase difference was not statistically significant (*p* = 0.78). Among the 9 patients with type 1 HAE-C1INH, mean C1 inhibitor antigen was 0.069 ± 0.029 g/L (reference 0.21–0.39 g/L), and median functional activity was 11.0% (IQR 4.3–13.5%; reference ≥ 68%). Patient 4, who had type 2 HAE-C1INH, had increased C1 inhibitor antigen of 0.70 g/L and reduced function of 6.3%. Serum C1q was within the normal range in all patients (range 163–222 mg/L; reference 159–233 mg/L), consistent with hereditary rather than acquired C1INH deficiency (Table 1).

### *SERPING1* variants

Sanger sequencing identified 10 distinct heterozygous *SERPING1* variants, each observed in a single family (Table 2).

**Table 2:** *SERPING1* variants identified in 10 unrelated patients with HAE-C1INH.

Patient	Region	DNA change	Protein change	ACMG criteria	Family data	Segregation evidence	Final classification
1	Exon 6	c.933_934insGGAA	p.Pro312Glyfs*17	PVS1, PM2, PP1, PP4	Mother symptomatic	Available	Likely pathogenic
2	Exon 7	c.1067T > A	p.Val356Glu	PM2, PP3, PP1, PP4	Daughter, uncle asymptomatic carriers	Partial	Likely pathogenic
3	Exon 7	c.1034G > A	p.Gly345Glu	PM2, PM1, PP3, PP4	No relatives tested	Not available	Likely pathogenic
4	Exon 8	c.1396C > T	p.Arg466Cys	PM1, PM2, PP3, PP4, PS4	Twin sons asymptomatic carriers with abnormal complement results	Partial	Pathogenic
5	Exon 2	c.6dup	p.Ser3Leufs*17	PVS1, PM2, PP3, PP4	No relatives tested	Not available	Likely pathogenic
6	Exon 8	c.1346T > A	p.Leu449Gln	PM1, PM2, PP3, PP1, PP4	Sister symptomatic; two daughters asymptomatic carriers	Partial	Likely pathogenic
7	Exon 8	c.1483G > A	p.Val495Ile	PM2, PP1, PP4	Mother symptomatic; nephew asymptomatic	Partial	Variant of uncertain significance
8	Exon 5	c.816_818delCAA	p.Asn272del	PM4, PM2, PP3, PP1, PP4	Mother symptomatic; daughter asymptomatic	Partial	Likely pathogenic
9	Exon 7	c.1121T > C	p.Leu374Pro	PM2, PP3, PP4	Father symptomatic (deceased)	Incomplete	Variant of uncertain significance
10	Exon 8	c.1358_1359insCTGG	p.Val454Trpfs*20	PVS1, PM2, PP4	Two sons asymptomatic carriers	Partial	Likely pathogenic

ACMG/AMP evidence codes were assigned based on population frequency, predicted variant effect, previous reports, phenotype specificity, and segregation data when available.

The variant spectrum included:

Frameshift variants (*n* = 3): c.933_934insGGAA (p.Pro312Glyfs*17), c.6dup (p.Ser3Leufs*17), and c.1358_1359insCTGG (p.Val454Trpfs*20).

Missense variants (*n* = 6): c.1067T > A (p.Val356Glu), c.1034G > A (p.Gly345Glu), c.1396C > T (p.Arg466Cys), c.1346T > A (p.Leu449Gln), c.1483G > A (p.Val495Ile), and c.1121T > C (p.Leu374Pro).

In-frame deletion (*n* = 1): c.816_818delCAA (p.Asn272del).

All variants were absent from or extremely rare in gnomAD v4.1. According to ACMG/AMP criteria, c.1396C > T was classified as pathogenic; seven variants were classified as likely pathogenic; and two, c.1483G > A and c.1121T > C, were classified as variants of uncertain significance (VUS).

Co-segregation information was available for 8 of the 10 families (Table 2), providing supportive evidence for variant interpretation in several families.

### Long-term prophylaxis with lanadelumab

Seven patients (Patients 2, 4, 5, 6, 7, 8, and 10) received lanadelumab for LTP. Specifically, four patients (Patients 5, 7, 8, and 10; 57%) achieved complete attack-free status and had intervals extended to q6w (Patients 5, 7, 8) or q4w (Patient 10). Two additional patients (Patients 2 and 4; 29%) achieved well-controlled disease with only sporadic mild breakthrough attacks (AAR <1 per year) and had intervals extended to q8w (Patient 2) and q10w (Patient 4). Patient 6 (14%), despite achieving AECT ≥13 (well-controlled), experienced one mild abdominal breakthrough attack. Overall, interval extension ranged from q4w to q10w at the most recent follow-up (Table 3).

**Table 3:** Lanadelumab prophylaxis regimens and clinical outcomes in seven patients with HAE-C1INH.

Patient	Lanadelumab regimen and exposure period	Breakthrough attacks during prophylaxis	Attack-free status
2	(1) 2024-04-20 to 2025-05-17: lanadelumab 300 mg, q2w; (2) 2025-06-14 to 2025-08-11: lanadelumab 300 mg, q4w; (3) 2025-09-10 to 2025-11-06: lanadelumab 300 mg, q6w; (4) 2026-01-02 to present: lanadelumab 300 mg, q8w	Multiple mild attacks; all resolved spontaneously	Attack-free after last breakthrough attack since Aug 17, 2025
4	(1) 2023-11-20 to 2025-08-15: lanadelumab 300 mg, q4w; (2) 2025-09-13 to 2025-11-17: lanadelumab 300 mg, q8w; (3) 2026-01-15 to present: lanadelumab 300 mg, q10w	One mild hip swelling episode; resolved within 2 h	Attack-free after last breakthrough attack since Jul 14, 2024
5	(1) 2024-04-25 to 2024-12-24: lanadelumab 300 mg, q2w; (2) 2025-01-22 to 2025-07-23: lanadelumab 300 mg, q4w; (3) 2025-08-22 to present: lanadelumab 300 mg, q6w	None	Attack-free throughout
6	(1) 2024-06-21 to 2024-12-14: lanadelumab 300 mg, q2w; (2) 2024-12-15 to 2025-02-13: lanadelumab 300 mg, q4w; (3) 2025-03-15 to 2025-09-25: lanadelumab 300 mg, q6w; (4) 2025-11-19 to present: lanadelumab 300 mg, q8w	One mild abdominal attack; resolved within 1 h	Attack-free after last breakthrough attack since Feb 11, 2025
7	(1) 2024-09-15 to 2024-12-14: lanadelumab 300 mg, q2w; (2) 2024-12-30 to 2025-06-27: lanadelumab 300 mg, q4w; (3) 2025-07-30 to present: lanadelumab 300 mg, q6w	None	Attack-free throughout
8	(1) 2024-10-05 to 2025-04-06: lanadelumab 300 mg, q2w; (2) 2025-05-02 to 2025-11-01: lanadelumab 300 mg, q4w; (3) 2025-12-24 to present: lanadelumab 300 mg, q6w	None	Attack-free throughout
10	(1) 2025-05-13 to 2025-12-08: lanadelumab 300 mg, q2w; (2) 2025-12-30 to present: lanadelumab 300 mg, q4w	None	Attack-free throughout

Lanadelumab was initiated at 300 mg subcutaneously every 2 weeks in all treated patients. Dose intervals were subsequently extended according to individual disease control after shared decision-making. “None” indicates no breakthrough attacks during lanadelumab prophylaxis. q2w, every 2 weeks; q4w, every 4 weeks; q6w, every 6 weeks; q8w, every 8 weeks; q10w, every 10 weeks.

### Effect on attack frequency and disease control

Changes in AAR and AECT scores are summarized in Table 4.

**Table 4:** Changes in annualized attack rate and Angioedema Control Test scores before and after lanadelumab prophylaxis.

Patient	HAE	AAR (attacks/year)		AECT score	
		Baseline (12 months before treatment)	Post-treatment (during lanadelumab prophylaxis)	Baseline (3 months before treatment)	Post-treatment (most recent 3 months)
2	HAE-C1INH type 1	23	6	2	12
4	HAE-C1INH type 2	8	1	4	13
5	HAE-C1INH type 1	27	0	2	13
6	HAE-C1INH type 1	12	1	3	13
7	HAE-C1INH type 1	6	0	4	13
8	HAE-C1INH type 1	7	0	4	13
10	HAE-C1INH type 1	11	0	3	14
Median (IQR)		11 (7–23)	0 (0–1)	3 (2–4)	13 (13–13)
*P*-value			0.016		0.016

Data are presented as individual patient values or median (IQR). Baseline AAR refers to the 12 months before lanadelumab initiation, and post-treatment AAR was annualized according to each patient’s duration of lanadelumab exposure. Baseline AECT refers to the 3 months before lanadelumab initiation, and post-treatment AECT refers to the most recent 3 months. Paired baseline and post-treatment values were compared using the exact two-sided Wilcoxon signed-rank test. *P*-values were interpreted descriptively because of the exploratory nature of the study and limited sample size. AAR, annualized attack rate; AECT, angioedema control test; HAE, hereditary angioedema; HAE-C1INH, hereditary angioedema due to C1 inhibitor deficiency; IQR, interquartile range.

Among the 7 patients receiving lanadelumab, median AAR decreased from 11 (IQR, 7–23) to 0 (IQR, 0–1) attacks/year (*p* = 0.016). Median AECT score increased from 3 (IQR, 2–4) to 13 (IQR, 13–13) (*p* = 0.016). Four of seven patients (57%) remained completely attack-free during the observation period. Three patients experienced mild breakthrough attacks during lanadelumab prophylaxis: Patient 2 experienced multiple mild attacks, all of which resolved spontaneously; Patient 4 experienced one mild breakthrough attack with hip swelling, which resolved within 2 h; and Patient 6 experienced one mild abdominal attack, which resolved within 1 h. No breakthrough attacks required acute treatment. All seven patients achieved AECT scores ≥10 during treatment, indicating well-controlled disease.

### Safety

Lanadelumab was well tolerated. No serious adverse events, thromboembolic events, or treatment discontinuations occurred. No clinically significant injection-site reactions were recorded.

Duration of lanadelumab exposure varied across the cohort according to treatment initiation dates. The seven patients receiving lanadelumab had a median follow-up duration of 18 months (range, 12–26 months) as of May 2026. Specifically, Patient 2 had the longest exposure (26 months; 2.2 years), Patient 4 had 30 months (2.5 years), Patient 5 had 25 months (2.1 years), Patient 6 had 23 months (1.9 years), Patient 7 had 20 months (1.7 years), Patient 8 had 19 months (1.6 years), and Patient 10 had 12 months of follow-up. All patients remained on treatment at the time of manuscript preparation, with no discontinuations due to adverse events or loss of efficacy.

### Acute treatment with icatibant

Patient 9 received subcutaneous icatibant 30 mg for an acute abdominal attack. Symptom relief was achieved within 30 min, and the attack resolved completely within 2 h.

### Patients not receiving long-term prophylaxis

Three patients did not receive LTP. Patient 1, who had comorbid obsessive-compulsive disorder, experienced reduced attack frequency after optimization of psychotropic medications and elected to defer LTP. Patient 3 had infrequent, mild attacks and declined prophylaxis after shared decision-making. Patient 9 declined both attenuated androgens and lanadelumab but retained access to on-demand icatibant for acute attacks.

## Discussion

In this single-center retrospective observational study of 10 unrelated Chinese index patients with HAE-C1INH, we observed long diagnostic delays, frequent misdiagnosis before confirmation, marked heterogeneity of *SERPING1* variants, and improved disease control among patients receiving lanadelumab prophylaxis.

### Diagnostic delay and misdiagnosis

The mean diagnostic delay in this cohort was 9.4 years, with the longest delay reaching 23 years. This finding is consistent with previous Chinese and international reports showing substantial delays in the diagnosis of HAE-C1INH (20, 32). In the present series, abdominal attacks were often mistaken for pancreatitis, appendicitis, or gastroenteritis, whereas cutaneous swelling was misdiagnosed as allergic angioedema or chronic urticaria. One patient required emergency tracheostomy before HAE was diagnosed.

These findings indicate that HAE-C1INH should be considered in patients with recurrent angioedema without wheals, particularly when attacks are non-pruritic, poorly responsive to antihistamines or corticosteroids, or accompanied by recurrent abdominal pain or laryngeal symptoms. Earlier testing of serum C4 and C1 inhibitor antigen and function in emergency, gastroenterology, dermatology, otolaryngology, and allergy settings may help reduce diagnostic delay (9, 21, 30).

### Clinical phenotype and laboratory diagnosis

The clinical manifestations in this cohort were broadly consistent with previous reports of HAE-C1INH (6, 33, 34). All patients had peripheral edema, 70% had abdominal attacks, and 60% had facial or laryngeal involvement. The relatively high proportion of laryngeal involvement may reflect referral bias to a tertiary center, but it also underlines the need for timely diagnosis and access to on-demand treatment, as untreated laryngeal attacks can be fatal (35).

Reported triggers included emotional stress, fatigue, cold exposure, menstruation, minor trauma, and prolonged standing, consistent with known precipitating factors in HAE-C1INH (36). The role of hormonal factors as triggers could not be assessed in this small cohort.

All patients had reduced C4 levels during attacks. C4 remains a useful first-line screening test, but normal C4 outside attacks does not exclude HAE-C1INH when clinical suspicion is high (9, 37, 38). Therefore, C1 inhibitor antigen and functional testing remain essential for diagnosis. Normal C1q levels in all patients supported hereditary rather than acquired C1 inhibitor deficiency (9, 39). Consistent with this, anti–C1INH antibody testing was reserved for the two sporadic cases without a family history and was not performed routinely, because a positive family history together with normal C1q made acquired deficiency clinically improbable in the remaining patients.

### *SERPING1* variant spectrum

Ten distinct heterozygous *SERPING1* variants were identified in 10 unrelated families, with no recurrent variant. This finding is consistent with the marked allelic heterogeneity of HAE-C1INH (8, 13, 14). The variant spectrum included frameshift variants, missense variants, and one in-frame deletion.

Frameshift variants are expected to cause loss of function, most likely through nonsense-mediated mRNA decay or production of truncated non-functional protein (7, 8). The c.1396C > T variant in Patient 4 was associated with HAE-C1INH type 2, consistent with previous genotype–phenotype observations for variants affecting the reactive center loop (8, 40). The c.1067T > A variant identified in Patient 2 has also been reported in a Chinese patient with type 1 HAE-C1INH (41). Other missense variants were interpreted according to population frequency, in silico predictions, phenotype compatibility, and available segregation evidence.

Two variants, c.1483G > A and c.1121T > C, were classified as variants of uncertain significance. Both variants were detected in patients with typical clinical features and compatible complement abnormalities. However, they should not be considered disease-causing without additional segregation or functional evidence. Because functional validation was unavailable and segregation evidence was limited, these variants should be interpreted cautiously and re-evaluated when additional genetic or functional evidence becomes available.

These findings also highlight the genetic heterogeneity of HAE-C1INH and the potential value of comprehensive genetic evaluation in selected patients, particularly sporadic cases and families requiring cascade screening. Broader testing may be considered when conventional exon-based sequencing is negative or when clinical and genetic findings are discordant (15–18).

### Lanadelumab prophylaxis

Lanadelumab prophylaxis was associated with a marked reduction in attack frequency and improved disease control in this cohort. Four of the seven treated patients remained attack-free during follow-up, and the remaining three experienced only mild breakthrough attacks during lanadelumab prophylaxis, none of which required acute treatment. No serious adverse events or treatment discontinuations were recorded.

These observations are consistent with the HELP trial program and real-world studies showing that lanadelumab substantially reduces attack frequency and improves disease control in patients with HAE-C1INH (22–29). The present findings add to the limited real-world evidence on lanadelumab use in Chinese patients (27). In this cohort, good disease control was maintained in several patients after individualized extension of dosing intervals, which ranged from every 4 to every 10 weeks at the last follow-up. However, interval extension was individualized rather than protocol-driven, and treatment duration varied among patients. Therefore, these findings should be interpreted as preliminary and should not be considered evidence supporting a specific dose-extension schedule. Prospective studies with predefined criteria for interval adjustment are needed.

During the study period (January 2022–May 2026), the therapeutic landscape for HAE-C1INH in China evolved substantially. Lanadelumab became available and represented the targeted first-line long-term prophylaxis option recommended per international guidelines (9, 21, 30). Before lanadelumab became available (before ∼2021), management in China relied primarily on non-targeted strategies, including FFP for acute attacks and attenuated androgens (danazol) for long-term prophylaxis; icatibant became available in some major centers but with limited accessibility and higher cost.

In the wider Chinese context, HAE remains substantially underdiagnosed. Although population-based prevalence estimates of ∼1.0–1.6 per 100,000 would predict roughly 14,000–22,000 affected individuals nationwide, only a few hundred to around one to two thousand patients have been formally identified, indicating a large diagnostic gap. Expertise and diagnostic capacity are concentrated in a small number of tertiary referral centers in major cities—most prominently Peking Union Medical College Hospital, which coordinates the national HAE registry and the Chinese HAE patient organization—together with a limited number of provincial referral centers such as ours. Complement and C1INH functional testing, on-demand icatibant, and lanadelumab are largely confined to these urban centers, whereas patients in rural and lower-tier settings face limited testing availability, longer referral pathways, and reduced access to targeted therapy. Reimbursement is expanding but remains geographically uneven. Strengthening the national registry, broadening cascade screening of affected families, and decentralizing diagnostic testing are therefore key priorities for reducing diagnostic delay and access inequity in China.

As of mid-2026, lanadelumab has become increasingly accessible through medical insurance coverage in major urban centers, though significant regional disparities in availability and reimbursement persist. For acute on-demand management, icatibant is now available as the targeted bradykinin B2 receptor antagonist in select hospitals, but remains more limited in distribution than FFP; FFP continues to be the most widely accessible acute treatment option across both urban and rural regions and hospital tiers. Attenuated androgens continue to be used in some centers as an alternative long-term prophylaxis option, typically where lanadelumab is unavailable, rather than as an add-on to lanadelumab. In our cohort, no patient received attenuated androgens in combination with lanadelumab; combining attenuated androgens with lanadelumab is not consistent with current clinical practice and would not be an appropriate treatment approach, as guideline-recommended targeted prophylaxis is intended to replace, rather than supplement, non-targeted androgen therapy.

In this single-center cohort (based at a tertiary teaching hospital in a provincial capital), lanadelumab was accessible and selected as the long-term prophylaxis strategy in 7 of 10 patients, with demonstrable efficacy and safety benefits. Three patients declined long-term prophylaxis for various reasons: Patient 1 experienced reduced attack frequency after optimization of psychotropic medications for comorbid obsessive-compulsive disorder and elected to defer LTP; Patient 3 had infrequent, mild attacks and declined prophylaxis after shared decision-making; and Patient 9 declined both attenuated androgens and lanadelumab but retained access to on-demand icatibant for acute attacks. These findings support the expanding use of targeted biological therapies in Chinese patients with HAE-C1INH, consistent with international guidelines recommending early intervention for high-risk patients. However, they also underscore the need for further expansion of treatment options, improved awareness among healthcare providers, and enhanced healthcare system readiness to support timely diagnosis and equitable access to advanced therapies across urban and rural regions and hospital tiers. Access barriers and regional heterogeneity remain significant challenges to optimizing HAE management in China.

### Acute treatment and psychological factors

One patient received icatibant for an acute abdominal attack and had symptom relief within 30 min and complete resolution within 2 h, consistent with the known efficacy of icatibant as a bradykinin B2 receptor antagonist for acute HAE attacks (42).

Patient 1 reported fewer attacks after optimization of treatment for obsessive-compulsive disorder. Emotional stress is a recognized trigger of HAE attacks (36), and psychosocial burden is common in patients with HAE (43, 44). This single observation does not establish causality, but it suggests that psychological assessment and stress management may be useful components of care in selected patients.

A previous Chinese report has described lanadelumab prophylaxis, regional genetic diagnosis, and individualized management of HAE-C1INH (45). The present study extends these observations by integrating diagnostic delay, complement phenotyping, *SERPING1* variant classification, and real-world individualized lanadelumab prophylaxis outcomes in 10 unrelated index patients. This combined clinical-genetic-treatment perspective may provide additional insights into HAE-C1INH recognition and management in China.

### Limitations

This study has limitations. The small sample size and single-center retrospective design limit generalizability and may have introduced recall bias, particularly because some pre-treatment attack data were obtained through telephone follow-up. Follow-up duration and lanadelumab dosing intervals varied, and interval extension was individualized rather than protocol-driven; therefore, off-label extension beyond approved schedules should be interpreted cautiously. In addition, comparisons of AAR and AECT scores were exploratory because only seven patients received lanadelumab, and *p*-values should be considered descriptive. Genetic analysis was limited to Sanger sequencing of the *SERPING1* promoter, coding exons, and flanking intronic regions, so large deletions or duplications, deep intronic variants, and variants in other HAE-associated genes may have been missed. Segregation analysis was incomplete in some families, and functional validation was unavailable for VUS. Despite these limitations, our findings provide additional real-world evidence on the diagnosis, *SERPING1* allelic heterogeneity, and individualized lanadelumab prophylaxis of HAE-C1INH in Chinese patients.

## Conclusion

This single-center retrospective observational study suggests that Chinese patients with HAE-C1INH may experience long diagnostic delays and frequent misdiagnosis before confirmation. Ten distinct *SERPING1* variants were identified in 10 unrelated families, supporting the marked genetic heterogeneity of the disease. Lanadelumab prophylaxis was associated with reduced attack frequency, improved AECT scores, and good tolerability in this small real-world cohort. Earlier complement testing, appropriate genetic analysis, and access to both on-demand and prophylactic therapies may improve the care of Chinese patients with HAE-C1INH. Larger prospective multicenter studies are needed to clarify genotype–phenotype correlations and optimize individualized long-term prophylaxis strategies.

## Data Availability

The datasets presented in this study can be found in online repositories. This study identified 10 SERPING1 variants. Five variants have been submitted to and are listed in ClinVar (https://www.ncbi.nlm.nih.gov/clinvar/), with Variation IDs of 426682, 2137099, 2137097, 3947, and 4857361. The other five variants have been submitted to and are listed in LOVD (https://databases.lovd.nl/shared/variants/SERPING1), with variant accession identifiers of Variant #0001078360, Variant #0001044610, Variant #0001021668, Variant #0000971694, and Variant #0001059325.
